# Innovative Strategies Toward the Disassembly of the EPS Matrix in Bacterial Biofilms

**DOI:** 10.3389/fmicb.2020.00952

**Published:** 2020-05-26

**Authors:** Rita M. Pinto, Filipa A. Soares, Salette Reis, Cláudia Nunes, Patrick Van Dijck

**Affiliations:** ^1^LAQV, REQUIMTE, Departamento de Ciências Químicas, Faculdade de Farmácia, Universidade Do Porto, Porto, Portugal; ^2^Laboratory of Molecular Cell Biology, Institute of Botany and Microbiology, KU Leuven, Leuven, Belgium; ^3^Center for Microbiology, VIB-KU Leuven, Leuven, Belgium

**Keywords:** bacterial resistance, matrix disruptive agents, nanocarriers, photodynamic therapy, ultrasounds, magnetic nanoparticles

## Abstract

Bacterial biofilms represent a major concern at a worldwide level due to the high demand for implantable medical devices and the rising numbers of bacterial resistance. The complex structure of the extracellular polymeric substances (EPS) matrix plays a major role in this phenomenon, since it protects bacteria from antibiotics, avoiding drug penetration at bactericidal concentrations. Besides, this structure promotes bacterial cells to adopt a dormant lifestyle, becoming less susceptible to antibacterial agents. Currently, the available treatment for biofilm-related infections consists in the administration of conventional antibiotics at high doses for a long-term period. However, this treatment lacks efficiency against mature biofilms and for implant-associated biofilms it may be necessary to remove the medical device. Thus, biofilm-related infections represent an economical burden for the healthcare systems. New strategies focusing on the matrix are being highlighted as alternative therapies to eradicate biofilms. Here, we outline reported matrix disruptive agents, nanocarriers, and technologies, such as application of magnetic fields, photodynamic therapy, and ultrasounds, that have been under investigation to disrupt the EPS matrix of clinically relevant bacterial biofilms. In an ideal therapy, a synergistic effect between antibiotics and the explored innovated strategies is aimed to completely eradicate biofilms and avoid antimicrobial resistance phenomena.

## Introduction

A post-antibiotic era is now emerging due to the increasing figures of antimicrobial resistance cases at a worldwide level. This phenomenon occurs naturally, however the use and misuse of antimicrobial agents in humans and animals promoted its acceleration in the last decades (WHO, 2014).

Bacterial biofilms are key players in the development of antimicrobial resistance. Biofilms are formed when bacterial cells attach to a substratum or to other cells embedded in a protective polymeric extracellular matrix (Pinto et al., 2019). The biofilm formation process can be divided in three main stages: attachment, maturation, and detachment. As soon as a medical device is implanted in the human body, host matrix proteins immediately adhere to the implant surface. Bacterial cells are able to recognize and attach to these proteins, promoting bacterial colonization. From this point, the biofilm grows by formation of a matrix of extracellular polymeric substances (EPS) around bacterial cells until it reaches a phase of maturation, adopting a three-dimension structure (Pinto et al., 2019). Eventually, environmental stimuli may lead to the detachment of single cells or cell clusters from the biofilm, promoting dissemination and colonization on other sites of the host (Beitelshees et al., 2018; Pinto et al., 2019).

In biofilm communities, the EPS matrix is responsible for intercellular interactions and protection of bacterial cells from hostile environment. Thus, this matrix mainly contributes to the increased antibiotic tolerance and resistance of biofilms compared with planktonic cells (Flemming et al., 2016; Fulaz et al., 2019). This review provides an overview of the characteristics of the EPS matrix and its role in antibiotic resistance. Besides, biofilm-associated diseases and innovative therapeutic strategies to disrupt the biofilm matrix are also outlined.

## The Biofilm Matrix and Antimicrobial Resistance

**Figure 1:**
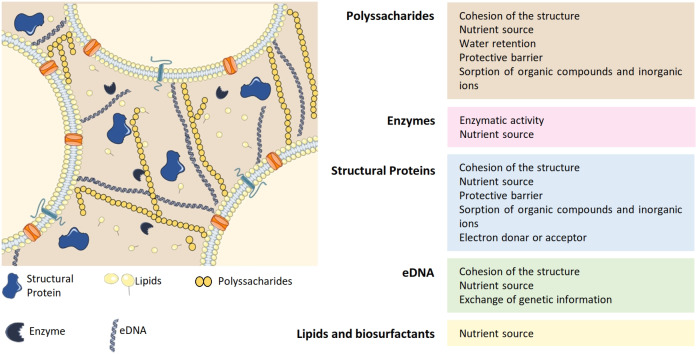
Composition of the EPS matrix and the functions of its major constituents: polyssacharides, enzymes, structural proteins, eDNA, lipids, and biosurfactants. Adapted from Flemming and Wingender (2010), Koo et al. (2017). eDNA, extracellular DNA.

The formation of the biofilm matrix is a dynamic process with high energetic cost for bacteria since it requires production and secretion of extracellular material. In exchange, the EPS matrix provides mechanical stability to the biofilms and mediates interactions between cells (Flemming et al., 2016). In most cases, the biofilm matrix represents around 90% of the total biofilm biomass and is mainly composed by polysaccharides, lipids, proteins and extracellular DNA (eDNA) (Fulaz et al., 2019) (Figure 1). Extensive reviews regarding biofilm matrix and antimicrobial resistance can be found elsewhere (Donlan and Costerton, 2002; Flemming and Wingender, 2010).

Polysaccharides are one of the main constituents of the EPS matrix and they attach to cell surfaces forming a complex network. Most of these molecules are heteropolysaccharides composed by a mixture of neutral and charged sugar residues. Additionally, they can contain organic and inorganic substituents, which contributes to their polyanionic or polycationic nature. The exopolysaccharides composition may differ between species and even between strains from the same species (Flemming and Wingender, 2010). Despite the heterogeneity among biofilms, exopolysaccharides are indispensable to biofilm formation and constitute the protective barrier of the EPS matrix (Flemming and Wingender, 2010; Fulaz et al., 2019). Besides, they are also responsible for water retention within the biofilm (Fulaz et al., 2019). Due to the high amount of water, the biofilm provides a highly hydrated environment that protects cells from fluctuations in water potential. In addition, the presence of water confers the biofilm a non-rigid structure with different viscosities that allow movement of the cells in the matrix (Flemming et al., 2016). As a result, a biofilm is a porous structure with macrocolonies surrounded by water-filled voids (Donlan, 2001a).

Extracellular proteins, such as structural proteins and enzymes, are also critical components of the matrix and can even be present in a higher amount than polysaccharides. Structural proteins are mainly involved in stabilization of the biofilm architecture, by connecting cells to the EPS (Fong and Yildiz, 2015). Enzymes are essentially involved in the degradation of other matrix components such as polysaccharides (e.g., dispersin B), matrix proteins (e.g., proteases), and eDNA (e.g., DNases). Thus, enzymatic activity within the biofilm provides nutrients to bacterial cells and promotes biofilm reorganization and dispersal (Fong and Yildiz, 2015).

Besides polysaccharides and proteins, eDNA also contributes for the structural integrity of the matrix (Fulaz et al., 2019). The contribution of this component for the three-dimensional structure of the biofilm differs greatly among species (Beitelshees et al., 2018). For instance, eDNA is a major component of *Pseudomonas aeruginosa* (*P. aeruginosa*) biofilms, while it is found in very low amounts in *Staphylococcus epidermidis* (*S. epidermidis*) biofilms (Beitelshees et al., 2018). Besides supporting the biofilm structure, eDNA facilitates exchange of genetic information between bacterial cells within the biofilm (Flemming and Wingender, 2010).

The complex nature of the matrix represents a diffusion barrier for antimicrobial agents, since it limits their penetration into deeper layers of the biofilm (Donlan and Costerton, 2002; Srivastava and Bhargava, 2016). Besides, within the matrix, antibiotics can interact with EPS components, leading to a decrease of their activity due to enzymatic degradation, complex formation owing to chelation, among other reactions (Flemming et al., 2016). The existence of various biofilm microenvironments, with different physical features such as low oxygen and pH, also influence the efficiency of antibacterial agents (Srivastava and Bhargava, 2016). Therefore, antimicrobial agents usually reach bacteria at sublethal concentrations, which boosts selection of antimicrobial resistance in the biofilm cells (Flemming et al., 2016).

In addition, bacterial cells embedded in the biofilm structure behave differently than in the planktonic state (Donlan and Costerton, 2002). In the lower regions of the biofilm, bacteria adopt a dormant lifestyle, since they have reduced access to nutrients and gaseous exchange (Flemming et al., 2016). These cells are metabolically less active than planktonic cells, leading to their reduced susceptibility to antibiotics (Anderson and O'Toole, 2008). This resistance may lead to gene modification that can be transferred to other bacteria, through the facilitated intercellular communication promoted by the EPS matrix (Flemming et al., 2016). Thus, this phenomenon also contributes to the enhanced antibacterial resistance of biofilms.

Due to its complex composition and structure, the EPS matrix has a major role in the biofilm formation, development and survival. It is not only a protective barrier against external factors, but also a source of nutrients and enzymes, and an intercellular connector. Ultimately, the unique characteristics of the matrix contributes to the high antimicrobial tolerance and/or resistance of biofilms.

## Biofilms in Medical Devices and in Disease

**Figure 2:**
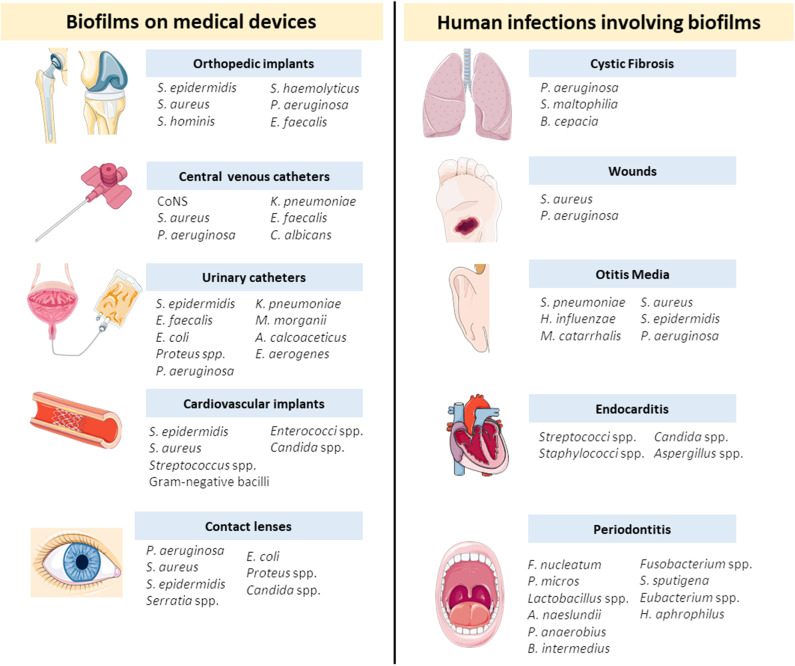
Biofilms associated to medical devices and chronic diseases and the most prevalent microorganisms for each device or disease (Donlan and Costerton, 2002; Stoica et al., 2017; Del Pozo, 2018). *A. calcoaceticus, Acinetobacter calcoaceticus*; *A. naeslundii, Actinomyces naeslundii*; *B. cepacia, Burkholderia cepacia*; *B. intermedius, Byrrhodes intermedius*; *C. albicans, Candida albicans*; CoNS, Coagulase-negative staphylococci; *E. coli, Escherichia coli*; *E. aerogenes, Enterobacter aerogenes*; *E. faecalis, Enterococcus faecalis*; *F. nucleatum, Fusobacterium nucleatum*; *H. aphrophilus, Haemophilus aphrophilus*; *H. influenzae, Haemophilus influenzae*; *K. pneumoniae, Klebsiella pneumoniae*; *M. catarrhalis, Moraxella catarrhalis*; *M. morganii, Morganella morganii*; *P. aeruginosa, Pseudomonas aeruginosa*; *P. anaerobius, Peptostreptococcus anaerobius*; *P. micros, Peptostreptococcus micros*; *S. aureus, Staphylococcus aureus*; *S. epidermidis, Staphylococcus epidermidis*; *S. haemolyticus, Staphylococcus haemolyticus*; *S. hominis, Staphylococcus hominis*; *S. maltophilia, Stenotrophomonas maltophilia*; *S. pneumoniae, Streptococcus pneumoniae*; *S. spuntigena, Selenomonas sputigena*.

Nowadays, biofilms represent an enormous concern for healthcare systems due to the escalating figures of antimicrobial resistance events and the high demand for implantable medical devices (Archer et al., 2011). Several biofilm forming organisms are commonly associated to medical devices or to chronic infections such as cystic fibrosis, otitis media, and wounds. In Figure 2 there is a detailed list of the most common medical devices and chronic infections and a list of the most prevalent microorganisms for each medical case.

Medical devices are known to increase the life quality of patients at a world level, but they are frequently associated to infections. When a medical device is implanted in the human body, colonization by bacteria occurs and a biofilm community may be established (Del Pozo, 2018). Several devices currently in medical use are prone to biofilm development by single or multiple microorganisms (Del Pozo, 2018). Some of the concerning examples will be further explored.

The number of orthopedic implants for bone fixation and joint replacement have been increasing in the last decades (Zimmerli, 2014). These implants are crucial to improve the life quality of patients, however, they are frequently associated to infections with devastating consequences, such as chronic pain and immobility (Ribeiro et al., 2012). According to Trampuz and Widmer, nearly 5% of orthopedic implants are infected and the susceptibility for infection increases by 5–40% in the case of a further surgery (Trampuz and Widmer, 2006). The infection may be caused by direct contamination of the device or from the contaminated wound and is frequently associated to opportunistic microorganisms (Stoica et al., 2017). For instance, studies show that periprosthetic joint infections are mainly associated to *Staphylococcus aureus* (*S. aureus*) and coagulase-negative staphylococci, such as *S. epidermidis* (Ribeiro et al., 2012; Zimmerli and Sendi, 2017). Most of these infections are caused by a single species, with only 16% of the cases being prompted by a mixed community (Stoica et al., 2017). Unfortunately, infections related to orthopedic implants are difficult to treat and usually require debridement and eventually removal of the device (Pinto et al., 2019). Extensive reviews addressing orthopedic implant-associated infections can be found in the literature (Ribeiro et al., 2012; Zimmerli, 2014; Zimmerli and Sendi, 2017).

Catheters are also extensively used in medical practice. Among these, urinary catheters-associated infections are the most common to occur and are predominantly caused by *Escherichia coli* (*E. coli*) and species of the genera *Pseudomonas, Klebsiella, Enterobacter*, and *Candida* (Stoica et al., 2017). On the other hand, coagulase-negative staphylococci (e.g., *S. epidermidis*) and *S. aureus* are the bacterial species mainly found in central venous catheters (Donlan, 2001b).

Less common medical devices-associated infections are verified in cardiovascular implants, such as cardiac prosthetic valves and stents (Stoica et al., 2017). Despite less frequent, these infections are a huge concern due to the high mortality rate that can reach 30% of the patients (Darouiche, 2004).

Besides implant-related infections, biofilms also play a role in chronic diseases in the oral cavity, ear, gastrointestinal, and urinary tracts, wounds and airways, among others (Del Pozo, 2018). For instance, the colonization of the lower respiratory tract by *P. aeruginosa* may lead to a chronic disease, cystic fibrosis, which is characterized by the accumulation of pulmonary secretions. Biofilms associated to chronic wounds, such as diabetic foot ulcers, are currently a clinical burden to patients since it delays the healing process (Del Pozo, 2018). Infections in the middle ear are also very common, especially in childhood (Donlan and Costerton, 2002). Otitis media involves inflammation of the mucoperiosteal lining and the associated biofilms are difficult to treat due to the low penetration of antibiotics into the middle ear fluid. Another frequent condition involving biofilms is endocarditis, which develops when damaged endothelium interacts with bacteria or fungi in the bloodstream (Donlan and Costerton, 2002).

When a biofilm is established, cells or cell clusters may detach from the structure and colonize other sites, leading to new infections (Donlan and Costerton, 2002). Moreover, bacteria within biofilms produce endotoxins and are more resistant to the host immune system (Donlan and Costerton, 2002). Despite the high complexity of biofilm-associated infections, current therapies still consist in the administration of conventional antimicrobial agents at high doses for a long-term period (Koo et al., 2017; Pinto et al., 2019). Ultimately, these therapies lack efficiency since they fail to approach combinatory strategies that target more than one component of the biofilm microenvironment (Koo et al., 2017).

## New Strategies to Eradicate Bacterial Biofilms

**Figure 3:**
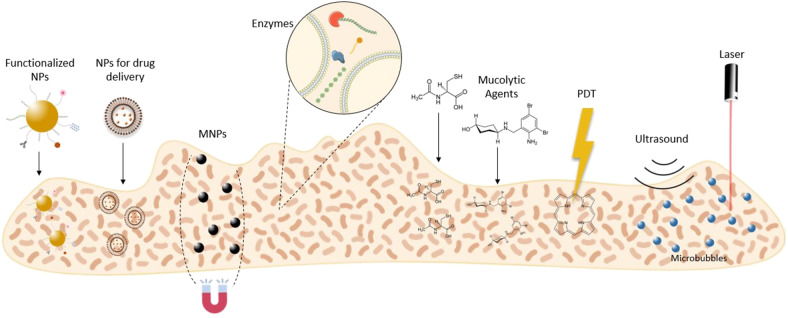
Innovative therapeutic strategies to disrupt the biofilm matrix. MNPs, magnetic nanoparticles; NPs, nanoparticles; PDT, photodynamic therapy.

The EPS matrix remains a critical challenge for bacterial biofilm eradicating strategies, due to its complexity and variability (Fulaz et al., 2019). Nevertheless, many research works are emerging in this field. Thus, this review is focused on innovative strategies to disrupt the EPS matrix of mature biofilms. Here, we outline reported disruptive agents, nanocarriers, and technologies for matrix disassembly (Figure 3).

### Matrix Disruptive Agents

The increasing resistance to antibiotics in combination with their lack of efficiency on biofilms are leading researchers to focus on the EPS matrix as a barrier to overcome. Thus, many studies in the literature highlight several antibiofilm agents capable of disrupting the EPS matrix in mature biofilms (Table 1).

**Table 1:** Biofilm disruptive strategies based on matrix disruptive agents.

**Strategy**	**Agent**	**Associated compound**	**Mechanism of action**	**Bacterial strains**	**Disease model**	**References**
Enzymes	**Dispersin B** (0–20 μg/sample)	-	Disrupt PNAG	*S. epidermidis* (strain 210) *B.cenocepacia* (*SCCH2:Bcn33- 1220 ST709*) *A. xylosoxidans* (*SCCH3:Ach 33-1365 glt allele 2*)	-	Dobrynina et al., 2015
	**Dispersin B** (0.03 U ml^−1^)	Ag-BP2 peptide	Disrupt PNAG	*S. epidermidis* (ATCC 35984)	-	Chen and Lee, 2018
	**DNase I** (5.0 μg ml^−1^)	Ampicillin Cefotaxime Rifampin Levofloxacin Azithromycin (50 × MIC)	Disrupt eDNA Antibacterial activity	*E. coli* (ATCC 25922) *H. influenzae* (VT 450-2006) *K. pneumoniae* (VT 1367) *P. aeruginosa* (ATCC 27853) *S. aureus* (ATCC 29213) *S. pyogenes* (VT 59) *A. baumannii* (VT 126)	-	Tetz et al., 2009
	Alginate lyase (15 U) DNase I (100 mg l^−1^)	Vancomycin (0.25 g l^−1^)	Disrupt EPS Antibacterial activity	*E. faecalis* (clinical isolates) *E. faecium* (clinical isolates)	Urinary tract infections	Torelli et al., 2017
	DNase I (0.5 μg ml^−1^) Marine bacterial DNase (0.5 μg ml^−1^)	**Essential oils from:** *Pogostemon heyneanus Cinnamomum tamala* 1 and 1.5% (v/v)	Target eDNA Antibacterial activity	MRSA (ATCC 33591)	-	Rubini et al., 2018
	Esperase® (8.3 × 10^−4^ U ml^−1^)	Prontosan® (10%) EDTA (10 mM)	Proteins cleavage Matrix disruption Antibacterial activity	*P. aeruginosa* (CIP 103.467) *S. aureus* (CIP 4.83)	Chronic wounds	Lefebvre et al., 2016
Mucolytic agent	Ambroxol (1.07 mg ml-1)	Ciprofloxacin (4.0 mg l^−1^)	Disrupt alginate Antibacterial activity	*P. aeruginosa* (PAO1)	Acute lung infection	Cheng et al., 2015
	Ambroxol (1.875 mg ml^−1^)	Vancomycin (2.0 and 5.0 mg ml^−1^)	Reduce EPS Antibacterial activity	*S. epidermidis* (ATCC 35984)	Catheter-related bloodstream infections	Zhang et al., 2015
	*N*-acetyl cysteine (12.5 mg ml^−1^)	-	Disrupt EPS	*S. epidermidis* (ATCC 12228) *P. acnes* (ATCC 6919) *M. smegmatis* (mc^2^ 155)	-	Eroshenko et al., 2017
	*N*-acetyl cysteine (0 – 100 mg ml^−1^)	-	Disrupt EPS	MRSA (clinical isolates) QRPA (clinical isolates)	Post-tympanostomy tube otorrhea	Jun et al., 2019
	*N*-acetyl cysteine (40 mg ml^−1^)	Linezolid (1.0 μg ml^−1^)	Disrupt EPS	*S. epidermidis* (clinical isolates: 9142 and 1457)	-	Leite et al., 2013

*A. baumannii, Acinetobacter baumannii; A. xylosoxidans, Achromobacter xylosoxidans; B.cenocepacia, Burkholderia cenocepacia; DNAse I, deoxyribonuclease I; E. coli, Escherichia coli; eDNA, extracellular DNA; EDTA, Ethylenediamine tetraacetic acid; E. faecalis, Enterococcus faecalis; E. faecium, Enterococcus faecium; EPS, extracellular polymeric substances; H. influenzae, Haemophilus influenzae; K. pneumoniae, Klebsiella pneumoniae; MIC, minimum inhibitory concentration; MRSA, Methicillin-resistant Staphylococcus aureus; M. smegmatis, Mycobacterium smegmatis; P. acnes, Propionibacterium acnes; P. aeruginosa, Pseudomonas aeruginosa; PNAG, poly-β(1-6)-N-acetylglucosamine; QRPA, quinolone-resistant Pseudomonas aeruginosa; S. aureus, Staphylococcus aureus; S. epidermidis, Staphylococcus epidermidis; S. pyogenes, Streptococcus pyogenes; U, unit*.

#### Enzymes

In a biofilm community, enzymes are naturally secreted by bacterial cells and retained within the matrix. Among other functions, enzymes are essential for the remodeling process of the biofilm. During this process, specific enzymes degrade components of the EPS matrix, leading to active dispersal of the biofilm. Consequently, the dispersed cells are able to recolonize other sites of the host (Flemming et al., 2016).

Despite being considered a biofilm virulence factor, these enzymes may be engineered to be used in strategies for biofilm disassembly. For instance, recombinant dispersin B was produced by cloning in *E. coli* a synthetic gene encoding the protein from *Aggregatibacter actinomycetemcomitans* (Dobrynina et al., 2015). After purification, the enzyme was tested against biofilms of *S. epidermidis, Burkholderia cenocepacia*, and *Achromobacter xylosoxidans*. Dispersin B actively disrupted mature *S. epidermidis* biofilms at low concentrations (lower than 0.3 μg per sample). However, for the other two strains, a dispersin B concentration above 5 μg per sample was required to reduce the biofilm biomass (Dobrynina et al., 2015). Dispersin B is a glycosyl hydrolase able to specifically disrupt poly-*N*-acetylglucosamine (PNAG), which is the main exopolysaccharide of *S. epidermidis* biofilms (Chaignon et al., 2007). Contrarily, PNAG is not a predominant component of *Burkholderia cenocepacia* and *Achromobacter xylosoxidans* biofilms, which may explain their higher resistance to the recombinant enzyme (Yakandawala et al., 2011; Dobrynina et al., 2015). In a more complex approach, Chen et al. conjugated recombinant dispersin B with a silver-binding peptide, which promotes *in situ* formation of silver nanoparticles (AgNPs) in the presence of silver ions (Lee et al., 2008; Chen and Lee, 2018). In this combinatory strategy, the recombinant enzyme disrupts the matrix and the AgNPs kills the dispersed cells (Chen and Lee, 2018). Comparing to dispersin B alone, the enzyme-peptide conjugate showed at least a 2-fold higher activity against 48 h-old *S. epidermidis* biofilms (Chen and Lee, 2018).

Besides dispersin B, deoxyribonuclease I (DNase I) has also been highlighted for antibiofilm purposes. The effects of DNase I in combination with several antibiotics were evaluated on 24 h-old biofilms (Tetz et al., 2009). The enzyme alone showed a 40% reduction in biofilm biomass for all tested strains. However, it did not compromise the number of viable cells. Antibiotics in combination with DNase I showed a decrease of 2- to 15-fold in the number of colony forming units (CFUs) in comparison with antibiotics alone. According to the authors, the disruption of eDNA by DNase I leads to a decrease of matrix material and, consequently, increases the efficiency of antibiotics (Tetz et al., 2009). Torelli et al. also reported the synergistic effect of matrix degrading enzymes and antibiotics. In this study, the efficacy of vancomycin in combination with DNase I or alginate lyase against 48 h-old biofilms was assessed (Torelli et al., 2017). For this purpose, three clinical isolates of *Enterococcus faecalis* (*E. faecalis*) and *Enterococcus faecium* (*E. faecium)* derived from catheter-associated urinary tract infections were used. In planktonic cells, both enzymes did not change the minimun inhibitory concentration (MIC) values of vancomycin. In *E. faecalis* biofilms, vancomycin alone, and vancomycin in combination with DNase I showed minimum biofilm eliminating concentration (MBEC) values of ~16 and 4 mg l^−1^, respectively. On the other hand, alginate lyase showed more potential than DNase I against *E. faecium* biofilms, with a MBEC reduction from ~16 mg l^−1^ (antibiotic alone) to 2 mg l^−1^ (vancomycin and alginate lyase).

In alternative to antibiotics, essential oils (EOs) derived from plants were also combined with DNases to promote the eradication of established biofilms (Rubini et al., 2018). EOs from *Pogostemon heyneanus* and *Cinnamomum tamala* were tested against mature methicillin-resistant *Staphylococcus aureus* (MRSA) biofilms alone and in combination with DNase I or marine bacterial DNase extracted from marine bacterium *Vibrio alginolyticus*. In *in vitro* efficacy studies, both DNase I and marine bacterial DNase in combination with EOs showed a percentage of inhibition around 85%. Through scanning electron microscopy (SEM) and an EPS quantification assay it was possible to verify a great reduction of EPS matrix after treatment with the EOs (Rubini et al., 2018). Hence, the combination of EOs with enzymes seems a promising strategy for biofilm eradication.

In a more complex approach, proteases were combined with antiseptics and ethylenediamine tetra-acetic acid (EDTA) for eradication of *S. aureus* and *P. aeruginosa* biofilms in chronic wounds (Lefebvre et al., 2016). EDTA is reported to destabilize the biofilm through cation chelation and inhibition of matrix metalloprotease activity (Raad et al., 2002; Percival et al., 2005). EDTA and enzymes can potentiate antiseptics activity, allowing the administration of lower doses (Lefebvre et al., 2016). Unlike the previous mentioned studies, Lefebvre et al. tested non-specific enzymes, with a broad-spectrum effect for several bacterial strains. The combinatory treatment revealed synergistic effects for both strains, with a significant reduction on bacterial viability. Nevertheless, the authors highlighted the need to develop a system able to efficiently deliver these molecules to the biofilm (Lefebvre et al., 2016).

Other enzymes, such as proteinase K (Kumar Shukla and Rao, 2013), glycoside hydrolases PelA_h_, and PslG_h_ (Baker et al., 2016), α-amylase (Kalpana et al., 2012; Watters et al., 2016), bromelain (Watters et al., 2016), lysostaphin (Watters et al., 2016), papain (Watters et al., 2016), and NucB (Shields et al., 2013) were investigated in a lesser extent for their ability to disrupt matrix components. Despite the promising *in vitro* efficacy of these enzymes, further studies in animal models are still required for validation purposes.

#### Compounds

Contrarily to enzymes, only few compounds have been reported to have active matrix disruptive properties. Among these, mucolytic agents, such as ambroxol and *N*-acetyl cysteine (NAC), are being highlighted for antibiofilm purposes.

Ambroxol is a frequently used mucolytic agent with antioxidant and anti-inflammatory effects in patients with pulmonary infections (Beeh et al., 2008; Paleari et al., 2011). Cheng et al. studied the combined effect of ambroxol with ciprofloxacin in a rat model of acute lung infection. With this aim, 3-days old biofilms of the mucoid strain *Pseudomonas aeruginosa* O1 (PAO1) were grown in the inner surface of inoculation tubes and further intubated in rats by intratracheal placement (Cheng et al., 2015). The rats were then treated once a day by inhalatory administration of antibiotic and ambroxol, for a total period of 7 days. The combination of ambroxol with ciprofloxacin showed lower bacterial counts compared to treatments with the antibiotic or the mucolytic agent alone. The biofilm morphology was assessed by SEM, where it was possible to observe thinner and less fibrous biofilms after treatment with the combination of ambroxol and the antibiotic. These results indicate that ambroxol has a synergistic effect with ciprofloxacin on mucoid biofilms. The authors speculate that this effect is a consequence of ambroxol enzymatic activity against alginate, which is the main component of these biofilms (Cheng et al., 2015). Ambroxol antibiofilm activity was also assessed against *S. epidermidis* biofilms in catheter-related bloodstream infections (Zhang et al., 2015). *In vitro* efficacy studies showed that ambroxol in combination with vancomycin significantly reduced bacterial viability and biofilm thickness, when compared with ambroxol or the antibiotic alone. By confocal laser scanning microscopy (CLSM) analysis, it was observable larger pore channels in the biofilm structure after treatment with ambroxol. Hence, these changes in the EPS matrix facilitates antibiotics penetration. Further *in vivo* studies in a rabbit infection model confirmed a synergistic effect between ambroxol and vancomycin, with disruption of mature biofilms and reduced inflammatory response (Zhang et al., 2015).

NAC is also a mucolytic agent with potential to eradicate bacterial biofilms. This synthetic agent is an antioxidant that disrupts disulfide bonds in mucus and inhibits cysteine use, by competition. Regarding to biofilms, NAC reduces production of EPS matrix and promotes disruption of mature structures (Romano et al., 2013). Several studies of NAC alone or in combination with antibiotics have been reported in the literature. For instance, Eroshenko et al. studied the effect of NAC on *S. epidermidis, Propionibacterium acnes*, and *Mycobacterium smegmatis* biofilms. The results showed that NAC has a high disruptive effect on a mixed biofilm of *S. epidermidis* and *Propionibacterium acnes*, with a 61% biomass reduction compared to the control. However, an insignificant reduction of biofilm biomass was verified when both strains were cultured alone, after a 4 h treatment with NAC (Eroshenko et al., 2017). More recently, the efficacy of NAC alone as an antibiofilm agent was evaluated in MRSA and quinolone-resistant *P. aeruginosa* biofilms, which are common in tympanostomy-tube infections (Jun et al., 2019). For both biofilm types, NAC showed a decrease of biofilm biomass comparing to the control group. The antibiofilm activity of NAC was further confirmed by a decrease in bacterial colonies and by a decrease in observable biofilm structure in SEM images (Jun et al., 2019). Some other studies also shown the synergy between NAC and antibiotics, such as fosfomycin and linezolid (Marchese et al., 2003; Leite et al., 2013).

Some compounds able to inhibit the production of EPS components, have also been reported. For instance, Siala et al. outlined the activity of the antifungal caspofungin acetate against MRSA biofilms. This lipopeptide acts in bacterial biofilms by inhibiting the PNAG synthesis. In combination with antibiotics, caspofungin acetate showed potential synergistic effects, both *in vitro* and in an animal model system for catheter-based infections (Siala et al., 2016). Besides this compound, other non-disruptive agents such as chitosan, *Cis*-2-decenoic acid (C_2_DA), nitric oxide, and rhamnolipids, may also trigger cell dispersion in bacterial biofilm (Chung and Toh, 2014; Fleming and Rumbaugh, 2017).

Although the promising results of the reported matrix disruptive agents, their therapeutic use still presents several limitations such as low bioavailability and non-specific biodistribution, which leads to adverse side effects and low concentrations at the target site (Poznansky, 1984). Hence, innovative vehicles for efficient delivery of enzymes and drugs to the target biofilms are a step forward in the design of new antibiofilm therapies.

### Nanocarriers

Nanosystems may play a critical role in both targeting and disruption of the EPS matrix. In the past few years, many researchers engineered sophisticated nanocarriers to increase penetration within the biofilm matrix and release their contents, such as antibiotics, closer to bacterial cells. This non-specific targeting is based on electrostatic interactions between the nanoparticles (NPs) and the matrix components (Fulaz et al., 2019).

The surface charge of NPs has an important role in the destruction of biofilms. The EPS matrix is manly composed by substances with a negative charge, including the bacterial cell wall. Thus, the EPS matrix is more likely to interact with positively charged NPs, which may lead to increased diffusion within the matrix comparing to neutral or negatively charged NPs (Fulaz et al., 2019). Several studies reported that lipid and polymer-based NPs showed increased efficacy against biofilms when positively charged (Lin et al., 2017; Thomsen et al., 2017; Wang et al., 2019). For instance, positively charged polymeric NPs were designed to bind and efficiently deliver nitric oxide to MRSA biofilms, for the treatment of diabetic wounds (Hasan et al., 2019). Gold NPs and nanotubes were also engineered to promote electrostatic interactions with the biofilm, by immobilizing the cationic polymer chitosan at the particles surface (Laskar et al., 2017; Lu et al., 2018; Khan et al., 2019). However, cationic NPs are considered more cytotoxic than neutral and negatively charged particles, which is a consequence of an enhanced cellular uptake (Frohlich, 2012; Hühn et al., 2013). Thus, Su et al. developed polymeric micelles containing polyurethanes with surface charge switchable properties due to protonation and deprotonation of tertiary amine groups in acidic and basic environments, respectively. Consequently, at the acidic pH of the biofilm, the micelles were able to switch to a positively charged surface, increasing their interaction with the matrix (Su et al., 2018). Mixed-shell polymeric micelles composed of the polyethylene glycol (PEG) and poly(β-amino ester) (PAE) were also developed for a pH-triggered switch of the surface charge (Liu et al., 2016). Electrostatic interactions may also be manipulated through the co-administration of negatively charged NPs with a penetration enhancer. Harper et al. observed that the co-administration of anionic alpha-tocopherol phosphate liposomes with a cationic electrolyte, Tris((hydroxymethyl)aminomethane), increased their penetration into the EPS matrix and interaction with bacteria. This phenomenon occurred due to ability of the cationic electrolyte to decrease the electrostatic interactions between the negatively charged liposomes and the biofilm components (Harper et al., 2019).

Targeting bacterial biofilms through electrostatic interactions and pH-triggered release, may offer appealing results for delivery of antimicrobial agents in higher concentrations to deeper layers of the biofilm. However, a complete physical removal of the biofilm is difficult to occur in biofilms of limited access (e.g., implant-related biofilms) (Flemming, 2011). Consequently, the remaining biofilm structures may provide an ideal site for colonization of other microbial cells. Additionally, mature biofilms contain dormant cells with higher resistance to antimicrobials, that may survive and recolonize the matrix (Flemming et al., 2016). Thus, a promising innovative approach consists in the addition of matrix disruptive enzymes and/or compounds, to the design of nanocarriers, in order to promote EPS disruption and dispersal of dormant cells (Table 2).

**Table 2:** Biofilm disruptive strategies based on nanodelivery systems.

**Strategy**	**Composition of the material**	**Physicochemical characteristics**	**Mechanism of action**	**Bacterial strains**	**Disease model**	**References**
Polymeric NPs	Chitosan TPP **Encapsulated compound:** Oxacillin **Functionalization:** DNase	**Size:** 166.7 nm **PDI:** 0.179 **Z. Potential:** +8.3 mV **LC:** 6.65%	Disrupt eDNA Electrostatic interactions Antibiotic controlled release	*S. aureus* (ATCC 6538)	-	Tan et al., 2018a
	PLGA PVA poly-L-lysine **Encapsulated compound:** Ciprofloxacin **Functionalization:** DNase I	**Size:** 251.9 nm **PDI:** 0.122 **Z. Potential:** + 28.9 ± 1.43 mV **Ciprofloxacin content:** 0.17 (w/w) (%)	Disrupt eDNA Antibiotic controlled release	*P. aeruginosa* (ATCC 15692) *S. aureus* (ATCC 12600)	Cystic fibrosis	Baelo et al., 2015
	Chitosan TPP **Functionalization:** DNase Cellobiose dehydrogenase	**Size:** 164.73 nm **Z. Potential:** + 13.07 mV	Disrupt eDNA Electrostatic interactions Antibacterial activity	*S. aureus* (ATCC 6538) *C. albicans* (DAY185)	-	Tan et al., 2020
	Chitosan TPP **Encapsulated compound:** Ciprofloxacin **Functionalization:** Alginate lyase	**Size:** 205.5 ± 9.0 nm **PDI:** 0.302 ± 0.031 **Z. Potential:** 12.2 ± 2.1 mV **EE:** 51.8 ± 2.1%	Disrupt extracellular alginate Antibacterial activity	*P. aeruginosa* (clinical isolate)	Cystic fibrosis	Patel et al., 2019
	Carboxymethyl chitosan Linolenic acid **Functionalization:** Dispersin B	**LE:** 51.14 ± 0.93% **LC:** 767.08 ± 13.90 mg g^−1^	Disrupt PNAG Electrostatic interactions	*S. aureus* (RN6390;15981; 8325; Col) *S. epidermidis* (QY301; RP62A; M187; 1457) *A. actinomycete mcomitans* (HK1651)	-	Tan et al., 2015
Gold NPs	Citrate-capped gold NPs **Functionalization:** Proteinase-K	**Size:** 27.17 ± 0.61 nm **PDI:** 0.238 ± 0.022 **Z. Potential:** −3.79 ± 0.21 mV	Disrupt extracellular proteins Antibacterial activity	*P. fluorescens* (PCL 1701)	-	Habimana et al., 2018
MOFs	gold MIL-88B (Fe) Cerium (IV) complexes	**LC:** 11.14 μ mol g^−1^	Target eDNA Peroxidase-like activity	*S. aureus* (ATCC 25923)	Topical wound	Liu et al., 2019

*A. actinomycetemcomitans, Actinobacillus actinomycetemcomitans; C. albicans, Candida albicans; DNase I, deoxyribonuclease I; eDNA, extracellular DNA; EE, encapsulation efficiency; LC, loading capacity; LE, loading efficiency; MOFs, Metal–organic frameworks; NPs, nanoparticles; P. aeruginosa, Pseudomonas aeruginosa; PDI, polydispersity index; P. fluorescens, Pseudomonas fluorescens; PLGA, poly(lactic-co-glycolic acid); PNAG, poly-β(1-6)-N-acetylglucosamine; PVA, poly(vinyl alcohol); S. aureus, Staphylococcus aureus; S. epidermidis, Staphylococcus epidermidis; TPP, Tri-poly phosphate; Z. Potential, Zeta Potential*.

With the purpose to combine antibacterial and antibiofilm agents in a nanocarrier, Tan et al. developed positively charged chitosan NPs co-encapsulating oxacillin and DNase I to eradicate 24 h-old *S. aureus* mature biofilms. A repeated treatment during 48 h revealed that NPs loading both the DNAse I and oxacillin exhibited higher antibiofilm activity than oxacillin-loaded NPs, with a 98.4% biofilm reduction. In addition, their positively charged surface facilitated penetration within the biofilm, without observable cytotoxicity effects against a human immortalized keratinocytes (HaCaT) cell line (Tan et al., 2018a). In a similar study, ciprofloxacin-loaded polymeric NPs were coated with DNase I covalently grafted to the cationic poly-L-lysine (Baelo et al., 2015). The results after a repeated treatment of 48 h-old *P. aeruginosa* biofilms for 3 days showed an eradication higher than 99.8%. The NPs safety profile was confirmed *in vitro* against J774 murine macrophages (Baelo et al., 2015). More recently, Tan et al. co-immobilized DNase I and cellobiose dehydrogenase in chitosan NPs to treat monomicrobial and polymicrobial biofilms of *Candida albicans* and *S. aureus*. Cellobiose dehydrogenase was selected as an antimicrobial agent since it uses cello-oligomers as a substrate to produce hydrogen peroxidase, which generates free radicals that promote oxidation of biofilm matrix components and has bactericidal effects (Henriksson et al., 2000; Gao et al., 2014; Tan et al., 2020). The efficiency of the developed NPs was tested in 24 h-old biofilms. The NPs revealed a high activity by reducing biofilm percentage more than 80% on both mono- and polymicrobial biofilms (Tan et al., 2020). NPs immobilizing only DNase I were also tested and showed no significant effect on the biofilms. According to the authors, this result is a consequence of the absence of a bactericidal agent, which allows dispersed bacterial cells to form a new biofilm. However, the co-immobilization with cellobiose dehydrogenase indicates a synergistic effect with DNase I (Tan et al., 2020).

The enzyme alginate lyase was also immobilized in polymeric NPs for matrix disruption purposes. Patel et al. designed ciprofloxacin-loaded NPs functionalized with alginate lyase for the treatment of biofilm-associated *P. aeruginosa* infection in cystic fibrosis. The *in vitro* efficacy assay against 48 h-old biofilms showed a complete disruption of the EPS matrix and no viable bacteria after repeated treatment for 72 h (Patel et al., 2019). This effect was not verified with antibiotic alone or in combination with alginate lyase and with non-functionalized NPs. Further, microscopy assessment of the biofilm confirmed the low biomass and biofilm thickness after treatment with the functionalized NPs. Additionally, *in vitro* and *in vivo* toxicity studies indicated a good biocompatibility of the developed nanosystem (Patel et al., 2019). In another study, Tan et al. designed chitosan NPs for immobilization of dispersin B from *Aggregatibacter actinomycetemcomitans* HK1651. The *in vitro* antibiofilm efficacy of this formulation was evaluated on *S. aureus, S. epidermidis*, and *Aggregatibacter actinomycetemcomitans* 24 h-old biofilms (Tan et al., 2015). For all tested strains, free and immobilized dispersin B showed a similar disruptive effect on the biofilms. Thus, the immobilization of the enzyme into polymeric NPs did not seem to compromise its activity (Tan et al., 2015).

Besides polymeric-based NPs, gold NPs were also functionalized with enzymes. Habimana et al. reported the synthesis of gold NPs functionalized with proteinase-K, combining bactericidal and matrix-degrading activities. The particles were tested against *Pseudomonas fluorescens* mature biofilms, showing a 78% thickness decrease comparing to the control (Habimana et al., 2018). However, this effect was similar to the non-functionalized particles (72% biofilm reduction).

Although the previous studies show a high potential of enzymes as antibiofilm therapies, their use is limited by high cost and poor stability (Wu et al., 2019). To overcome these issues, Liu et al. designed nanoenzymes based on metal organic frameworks (MOFs) and cerium (IV) complexes. In this system, MOFs have a peroxidase-like activity, which leads to bacterial cell death. On the other hand, cerium (IV) complexes mimic the DNase activity by hydrolyzing eDNA from 12 h-old *S. aureus* biofilms (Liu et al., 2019). *In vitro* efficacy studies showed the dispersal potential of the nanoenzyme. Nevertheless, the nanoenzyme alone was not able to have bactericidal activity. Hence, nanoenzymes were assessed in combination with free hydrogen peroxidase. In this condition, bacteria dispersed from the biofilm were efficiently killed. This result was further confirmed in *in vivo* studies using a subcutaneous model. Besides the antibiofilm effects, the treatment with the nanoenzyme and free hydrogen peroxidase revealed a significantly reduction of inflammation and negligible toxicity (Liu et al., 2019).

### Technologies for Biofilm Physical Removal

In the past few years, several technologies have been optimized to disrupt bacterial biofilms. These technologies are mainly based on magnetic field in association with NPs, phototherapy and ultrasounds.

#### Magnetic Field

Early studies based on the success of using magnetic and electric fields to affect other physiological processes triggered the hypothesis that this strategy could be effective in bacterial biofilm control, when in combination with appropriate antibiotics (Grosman et al., 1992; Khoury et al., 1992; Sinisterra, 1992; Benson et al., 1994). Therefore, in the last decades, association of magnetic fields with magnetic nanoparticles (MNPs), namely superparamagnetic iron-oxide nanoparticles (SPIONs) began to emerge. Among these, various magnetic field-based strategies to disrupt bacterial biofilms were reported (Table 3).

**Table 3:** Biofilm disruptive strategies based on magnetic field.

**Strategy**	**Method of exposure**	**NPs**	**Additional compound**	**Physicochemical characteristics**	**Bacterial strains**	**Mechanism of action**	**References**
External magnetic field	Static one-sided, Static switched, Oscillating,Static +oscillating	SPIONs (FluidMAGC-MX)	Ciprofloxacin loaded in spray-dried lactose particles	**Size:** 150 nm	*P. aeruginosa* (PAO1)	Disruption of the EPS matrix	Bandara et al., 2015
	AC and DC	Fe-oxide NPs coated with SiO_2_	–	**Sizes:** 8 nm, 11 nm, 70 nm	MRSA(ATCC 33592)	Damage and detachment of the matrix Magnetic hyperthermia	Li J. et al., 2019
	Static one-sided	Surface-modified SPIONs (CES, APTES, PEG functionalities)	-	*Bare*: **Size:** 13.7 ± 2.1 nm **-Zeta potential:** +43.7 ± 1.7 mV *CES-grafted*: **Size:** 13.8 ± 2.1 nm **Zeta potential:** −15.4 ± 0.5 mV *PEGylated*: **Size:** 14.9 ± 1.8 nm **Zeta potential:** −7.71 ± 0.9 mV *APTES- grafted*: **Size:** 17.8 ± 2.6 nm **Zeta potential:** +32.6 ± 0.3 mV	*S. aureus* (ATCC 12600) *S. epidermidis* (ATCC 35984)	Enhanced biofilm penetration Improved antibiotic efficacy	Subbiahdoss et al., 2012
	Static one-sided	SPIONs (Fe_3_O_4_)	Free Gentamicin	**Size:** 278 ± 61 nm	*S. aureus* (ATCC 12600; ATCC 5298)	Creation of Artificial Channels in the matrix	Quan et al., 2019
	Static one-sided	Fe_3_O_4_@Ag@HA	Gentamicin	**Zeta potential:** −19.4 mV **Saturation magnetization value:** 45.3 emu g^−1^	*S. aureus* (ATCC 25922)	ROS production Disruption and decomposition of the matrix Enhanced antimicrobial efficiency	Wang et al., 2018a
	Static one-sided	IOPs (Encapsulated SPIONs)	Encapsulated methicillin	*SPIONs* **Size:** 5 ± 2.5 nm *IOPs* **Size:** 83 ± 6 nm **Zeta Potential:** −1 ± 3 mV	*S. epidermidis* (RP62a)	Enhanced biofilm penetrationTargeted delivery	Geilich et al., 2017
	Static one-sided	Fe_3_O_4_@CS-PEG-Gent NPs	Gentamicin (loaded on the surface)	**Size:** ~40 nm **Zeta potential:** 8.7 mV	*S. aureus* (ATCC 5922)	Enhanced biofilm penetration Improved antibiotic efficacy	Wang et al., 2018b
	AC	SPIONs (Fe_3_O_4_)	Vancomycin	**Size:** 16 nm	*S. aureus* (BCRC10451)	Hyperthermia Improve antibiotic efficacy	Fang et al., 2017

*AC, alternative current; APTES, 3-aminopropyltriethoxysilane; CES, carboxyethylsilanetriol; CS, chitosan; DC, direct current; EPS, extracellular polymeric substances; Gent, gentamicin; HA, hyaluronic acid; IOPs, iron oxide polymersomes; MRSA, methicillin-resistant Staphylococcus aureus; NPs, nanoparticles; P. aeruginosa, Pseudomonas aeruginosa; PEG, polyethylene glycol; ROS, reactive oxygen species; S. aureus, Staphylococcus aureus; S. epidermidis, Staphylococcus epidermidis; SPIONs, superparamagnetic iron oxide nanoparticles*.

Several studies showed that, besides the good biocompatibility and low cytotoxicity, MNPs can be controlled and concentrated close to a target, through the application of an external magnetic field. This allows a deeper penetration into the biofilm and interferes in the organization of the matrix (Subbiahdoss et al., 2012; Bandara et al., 2015; Li J. et al., 2019; Quan et al., 2019). Consequently, possible elimination of the target biofilm is achieved through its breakup, along with cell detachment. Bandara et al. aimed to investigate the efficacy of different application modes of magnetic fields (static one-sided, static switched, oscillating, static + oscillating) in eliminating *in vitro* mature *P. aeruginosa* biofilms. These biofilms were treated with an aerosolized formulation containing different combinations of MNPs, ciprofloxacin and spray dried lactose (Bandara et al., 2015). Magnetic fields alone were able to disrupt the biofilms, negatively affecting the EPS matrix, either by interfering in its production or by direct disruption. Also, it was observed that the highest suppression of viability and biomass was achieved in biofilms exposed to a static switched field. The combination of static switched magnetic field with MNP/ciprofloxacin/MNP + ciprofloxacin showed the most promising results regarding the biofilm matrix disruption, which enables an easier penetration of the antibiotic to deeper layers of the biofilm (Bandara et al., 2015).

Besides magnetic targeting, MNPs also allow the increase in temperature by magnetic hyperthermia induction, through the application of alternating magnetic fields. Considering this additional effect, Li J. et al. compared the eradication efficiency of MNPs, with different sizes and concentrations, under AC and DC applied magnetic fields against MRSA biofilms. Greater cell detachment and matrix damage were observed for both 8 and 11 nm MNPs, exposed either to AC or DC magnetic fields. Considering all three tested sizes (8, 11, and 70 nm), although the application of AC fields allowed a local heating of the biofilms, DC fields showed to be the most effective strategy to break the EPS matrix and kill the bacteria. A 4.71 log_10_ reduction was achieved in biofilm bacteria after the treatment with 30 mg ml^−1^ of 11 nm NPs, under DC magnetic field (Li J. et al., 2019).

Although the type of the applied magnetic field and the size of MNPs are relevant, surface functionalities have also been suggested to be crucial in the interaction process with the EPS matrix and bacteria. Considering this, Subbiahdoss et al. showed that no differences in the antibiofilm efficacy were found between bare, carboxyethylsilanetriol(CES)-grafted and 3-aminopropyltriethoxysilane(APTED)-grafted SPIONs, against 24 h-old *S. epidermidis* biofilms. However, PEGylated SPIONs showed to be ineffective against staphylococcal biofilms. Optical cross-sections obtained using CLSM in the presence of magnetically concentrated CES-grafted SPIONs showed not only dead bacteria in the biofilm but also the formation of some channels across all the biofilm thickness (Subbiahdoss et al., 2012). In line with the previous findings, Quan et al. explored the mechanism behind the physical disruption of the biofilm matrix, responsible for the enhancement of the antimicrobial penetration. Similar to the results obtained by Subbiahdoss et al. (2012), formation of artificial channels, with around 1.4 μm of width, were observed in treated 24 h-old *S. aureus* biofilms. In addition, incubation of these biofilms with gentamicin caused a significant enhancement (4–6-fold) in staphylococcal killing, due to the improved penetration allowed by the non-biotical channels (Quan et al., 2019).

Other NPs have been added to this strategy in order to improve the antibiofilm effects of the magnetic field associated to MNPs. For instance, Wang et al. produced highly efficient nanoplatforms, consisting of gentamicin, tannic acid and AgNPs coated on MNPs, to test against established biofilms of *S. aureus*. Additionally to the MNPs expected effect, the presence of AgNPs caused the production of reactive oxygen species (ROS), which enhanced decomposition of polysaccharides and proteins of the EPS matrix (Wang et al., 2018a). Iron oxide-encapsulating polymersomes, containing both hydrophobic SPIONs and the hydrophilic antibiotic methicillin, were also developed to eradicate antibiotic-resistant infections associated with 24 h-old *S. epidermidis* biofilms (Geilich et al., 2017). It was proved that this formulation was able to completely eradicate all bacteria throughout the biofilm thickness, while not being toxic toward mammalian cells. Extensive bacterial death was observed within the boundaries of the magnetic field and SEM images of the biofilm ultrastructure showed both bacterial death and decoherence of the EPS matrix (Geilich et al., 2017). Wang et al. designed biocompatible Fe_3_O_4_/chitosan/PEG/Gentamicin MNPs aiming to eradicate mature *S. aureus* biofilms. This strategy combined the inner magnetic core and the loaded antibiotic, allowing the improvement of the effectiveness and bioavailability of gentamicin at acidic media, through the application of an external magnetic field. CLSM images showed that, just by applying the NPs to the biofilms, the biofilm structure starts to disrupt, and the number of dead bacteria significantly increases (Wang et al., 2018b). The proposed explanation for this phenomenon is the existence of electrostatic interactions between the positively charged MNPs and the negatively charged EPS matrix. Adding the external magnetic field, the biofilm matrix was found to be completely compromised, with an extensive bacterial death observed (Wang et al., 2018b). Also, dual-catalytic iron oxide MNPs have recently demonstrated to controllably kill, degrade, and remove biofilms. These NPs generate free radicals with bactericidal activity and promote EPS matrix degradation. Besides, a subsequent removal of the fragmented biofilm debris is achieved via magnetically forced nanoparticle movement (Hwang et al., 2019).

To evaluate the effect of MNPs-induced hyperthermia associated with antibiotics to treat osteomyelitis, Fang et al. mimicked a clinical situation of a chronic infection in an animal model. A metallic needle was implanted into the bone marrow cavity of distal femur of male Wistar rats, after the injection of *S. aureus*. The temperature increase, achieved through magnetic hyperthermia, led to biofilm destruction, without any significant damage on the surrounding tissues (Fang et al., 2017). The subsequent local administration of vancomycin into the femoral canal allowed biofilm eradication. Consistently with *in vitro* results, *in vivo* efficacy studies showed that MNPs under AC fields were able to compromise the protection barrier of biofilms through MNPs-induced hyperthermia, affecting their structure and enhancing the therapeutic effect of antibiotics (Fang et al., 2017).

#### Photodynamic Therapy and Ultrasounds

Photodynamic therapy (PDT) and ultrasound mediated therapies are also examples of technologies with growing interest due to their noninvasiveness, relatively low cost, flexibility, and minimal risk of inducing microbial resistance (Briggs et al., 2018). Both strategies, while being effective carried out separately, have already been combined, as further explored. PDT involves the use of nontoxic dyes, called photosensitizers (PS), which in the presence of molecular oxygen and visible light of appropriate wavelength can be excited, leading to the production of ROS (Hu et al., 2018). As a multiple target strategy, the generated ROS can not only oxidize several cellular components (e.g., lipids and DNA), leading to cell inactivation, but can also attack EPS molecules, causing the degradation of matrix structure (de Melo et al., 2013; Hu et al., 2018). Usually, highly conjugated, unsaturated organic molecules with large absorption coefficient in the visible spectrum can behave as PS (Hu et al., 2018). Considering these properties, a wide range of compounds of remarkably different structures have already been used as PS to target different types of biofilms (Table 4).

**Table 4:** Biofilm disruptive strategies based on photodynamic therapy.

**Strategy**	**PS and associated compounds**	**Light Parameters**	**Time of exposure**	**Bacterial strains**	**Mechanism of action**	**References**
PDT	TMP (10 μM) Vancomycin	*Tungsten lamp* **WL:** 400–800 nm(white light) **Power density:** 166 mW cm^−2^ **Fluence:** 150–200 J cm^−2^	~15–20 min	*S. aureus* (SA113, V329) MRSA (BH1C)	Bacteria dispersion Increased susceptibility to antibiotic	Di Poto et al., 2009
	ALA (40 mM)	*HPG5000 semiconductor laser* **WL:** 635 nm (red light) **Fluence:** 0–300 J cm^−2^	-	MRSA (ATCC 43300) MRSE (287)	Dose-dependent phototoxicity Interference in cell-to-cell and cell-to-matrix interactions	Li et al., 2013
	S-PS (0.5, 1, and 2 μg ml^−1^)EPIs	**WL:** 650 nm **Fluences:** 5, 10, 15 J cm^−2^	1 h	*S. aureus* (CMCC 260003)MDR *S. aureus* (ATCC 29213)	Morphological damage caused by ROS Enhanced antimicrobial efficiency	Jia et al., 2019
	ALA (10 or 20 mM)	*LED* **WL:** 630 nm(red light) **Power density:** 90 mW cm^−2^ **Fluence:** 108 J cm^−2^	20 min	*P. aeruginosa* (PAO1)	Dose-dependent growth inhibition and bacterial death Dispersion of the matrix	Tan et al., 2018b
	TBO, Azure A, and New MetB (10 μM)	**WL:** 630 nm **Power:** 100 mW **Power density:** 0.130 W cm^−2^ **Fluence:** 100 J cm^−2^	Maximum time exposure 13 min	*Enterococcus faecalis* (MTCC 2729) *Klebsiella pneumonia* (ATCC700603)	EPS disruption Reduction of EPS production	Misba et al., 2017
	Tetra-Py^+^-Me (20 μg)	*13 parallel OSRAM 2' 18 W/840 lamps* **WL:** 380–700 nm (white light) **Fluence:** Maximum of 64.8 J cm^−2^ **Power density:** 4.0 mW cm^−2^	Maximum time exposure 270 min	*S. aureus* (ATCC 700699) *P. aeruginosa* (57) *Candida albicans* (ATCC 10231)	Matrix decomposition (decrease of polysaccharides content)	Beirao et al., 2014
	TBO encapsulated in microemulsion(50 – 100 ppm)EDTA (100–500 ppm)	*LED lamps* **WL:** 610 – 630 nm(Red light) **Fluence:** 0.607 J cm^−2^	15 min	*S. aureus* (ATCC 35556) *S. epidermidis* (ATCC 35984)	EPS disruption by EDTA chelating effect Enhanced penetration of the PS	Rout et al., 2018
	ICG and EDTA(2 mM or 5 mM)Vancomycin for MRSAAmikacin for MRPA	*Diode laser* **WL:** 808 nm **Power density:** 1.5 W cm^−2^ **Fluence:** 135 J cm^−2^	90s	*S. aureus* (ATCC 25923)MRSA (10485) *P. aeruginosa* (ATCC 27853)MRPA (10911)	Formation of bacteria-free voids Bacterial death	Li X. et al., 2019
	Malachite greenconjugated to carboxyl-functionalized multi-walled carbon nanotubes(50 μg ml^−1^)	*Red Laser* **WL:** 660 nm **Fluence:** 58.49 J cm^−2^	3 min	*P. aeruginosa* (PAO1) *S. aureus*(MCC 2408)	Improved biofilm inhibition EPS inhibitionReduced cell viability	Anju et al., 2019b
	Surface Coating: IR780 (0.02 mg ml^−1^) MoS2 and arginine-glycine-aspartic acid-cysteine	**WL:** 808 nm **Power density:** 0.5 W cm^−2^	30 s intervals for 10 min	*S. aureus* (ATCC 29213)	Synergistic PDT/PTT effect(ROS generation/local hyperthermia)	Li M. et al., 2019
	ICG loaded into mesoporous polydopamine NPs	*Laser* **WL:** 808 nm **Power density:** 0.75 W cm^−2^ **Diameter:** 15.6 mm	600 s	*S. aureus* (ATCC 29213)	Synergistic PDT/PTT effect (ROS generation/local hyperthermia)	Yuan et al., 2019
	RLP068/Cl (50 μM)	*Diode laser* **WL:** 689 nm **Power density:** 120 mW cm^−2^ **Fluence:** 60 J cm^−2^	~8 min	MRSA (SAUMRBP2) *P. aeruginosa* (PAE2)	Decrease in biomass Decrease in the number of viable cells	Vassena et al., 2014
	MetB (0.3 mM)	*Laser* **WL:** 665 nm **Fluence:** 35 J cm^−2^ **Power density**: 35 mW cm^−2^	16 min	*S. aureus* MRSA *S. epidermidis* *P. aeruginosa* *A. baumannii* (sourced from NCTC and ATCC)	Bactericidal effect Total (or partial) eradication of formed biofilms	Briggs et al., 2018

*A. baumannii, Acinetobacter baumannii; ALA, 5-aminolevulinic acid; EDTA, ethylenediaminetetraacetic acid; EPIs, efflux pump inhibitors; EPS, extracellular polymeric substances; ICG, indocyanine green; LED, light-emitting diode; MDR S. aureus, multidrug-resistant Staphylococcus aureus; MetB, methylene blue; MRPA, multidrug-resistant Pseudomonas aeruginosa; MRSA, methicillin-resistant Staphylococcus aureus; MRSE, methicillin-resistant Staphylococcus epidermidis; NPs, nanoparticles; P. aeruginosa, Pseudomonas aeruginosa; PDT, photodynamic therapy; PS, photosensitizer; PTT, photothermal therapy; ROS, reactive oxygen species; S. aureus, Staphylococcus aureus; S. epidermidis, Staphylococcus epidermidis; S-PS, S-porphin sodium; TBO, toluidine blue O; TMP, tetra-substituted N-methyl-pyridyl-porphine; WL, wavelength*.

Di Poto et al. demonstrated the efficiency of tetra-substituted (N-methyl-pyridyl) porphine (TMP) as a PS on *S. aureus* mature biofilms. A TMP concentration of 10 μM and light doses of 150–200 J cm^−2^ allowed a significant decrease in survival of the biofilms (up to 2 log), with evident dispersion of the matrix and significant reduction in the number of adherent bacteria. Combining PDT with vancomycin resulted in 10^3^-10^4^ times lower counts than biofilm inhibitory concentrations used to kill untreated biofilms, showing that the detached and dispersed bacteria became more susceptible to antibiotics (Di Poto et al., 2009). Later, Li et al. revealed that 5-aminolevulinic acid (ALA) had potential to eliminate 24 h-old biofilms of MRSA and methicillin-resistant *S. epidermidis*. An increase of light dosage led to a gradual decrease in the survival as well as a decrease in the number of adherent bacteria in the biofilms of both strains (Li et al., 2013). After the highest dosage irradiation (300 J cm^−2^), SEM images showed a greater disruption of both biofilms, only with few aggregated colonies remaining. The mechanism of action could be associated with the loss of cell-to-cell and cell-to-matrix interactions, which led to dispersion of the bacterial cells, comprising the biofilm structure (Li et al., 2013). The same mechanism can also be used to explain the morphological damages observed in *S. aureus* and multidrug-resistant *S. aureus* biofilms, after a treatment with porphin sodium (Jia et al., 2019). For both strains, when a light dose of 15 J cm^−2^ was applied, a typical biofilm structure was no longer observed, being the adhesion between bacteria completely compromised (Jia et al., 2019).

Tan et al. also reported the effect of ALA-PDT but on 48 h-old *P. aeruginosa* biofilms. Regarding the biofilm structure, SEM images showed that, for 20 mM ALA complemented with a 108 J cm^−2^ light source treatment, the biofilm was visibly sparse, with bacteria presenting cracks, breaks, and different sizes (Tan et al., 2018b). In another study, phenothiazinium dyes (Toluidine blue O, Azure A, and New methylene blue) were tested against 24 h-old *E. faecalis* and *Klebsiella pneumoniae* preformed biofilms (Misba et al., 2017). A clear disruption of the EPS matrix was observed, comparing to the control groups, with 8log_10_ and 3log_10_ reductions in bacterial count achieved for *E. faecalis* and *Klebsiella pneumoniae*, respectively. Besides the disruptive effect on preformed biofilms, the results also showed an inhibition of EPS production in both species (Misba et al., 2017).

As previously referred, EPS matrix and specifically polysaccharides represent a huge portion of a biofilm. Thus, considering the reported effects of PDT on the matrix, Beirão et al. investigated the influence of a Tetra-Py^+^-Me-induced PDT on the extracellular polysaccharides of 24 h-old *P. aeruginosa* biofilms. In fact, a reduction of 81% on the polysaccharides was observed, which shows that these EPS components may be a primary target of photodynamic damage (Beirao et al., 2014).

Nevertheless, like other compounds, PS are also limited in their effectiveness due to the difficulty to penetrate the biofilm matrix as well as possible rapid degradation. Additionally, it is reported that some PS tend to aggregate in water, which also limits an effective therapy (Huang et al., 2010). To overcome these issues, different types of nanocarriers and nanostructures, such as chitosan NPs, gold NPs, carbon nanotubes, and silica NPs, have been developed to deliver and protect PS (Darabpour et al., 2016, 2017; Anju et al., 2019a; Paramanantham et al., 2019; Parasuraman et al., 2019; Mirzahosseinipour et al., 2020). Rout et al. studied the potential of Toluidine blue O in solution and in microemulsion, as well as Toluidine blue O in microemulsion with EDTA, in 16 h-old *S. aureus* and *S. epidermidis* biofilms. SEM and fluorescence microscopy results showed that the most effective treatment was the one done with encapsulated Toluidine blue O, with higher biofilm damaged, regarding its EPS matrix as well as bacteria viability (Rout et al., 2018). The combination of this formulation with EDTA resulted in an enhanced inhibition of bacterial biofilms, being ~100% for *S. aureus* biofilms and 80% for *S. epidermidis* biofilms. Surfactants and EDTA combined were able to penetrate deeper into the biofilms, due to the smaller microemulsion size achieved with the encapsulation of Toluidine blue O. Additionally, the chelating effect of EDTA allowed EPS matrix disruption, through sequestration of Ca^2+^ and Mg^2+^ ions, leading to an easier penetration (Rout et al., 2018). Considering this effect, recent studies also proposed a combination of PDT, EDTA, and antibiotics as an efficient approach to disrupt the matrix of 24 h-old MRSA and multidrug-resistant *Pseudomonas aeruginosa* (MRPA) biofilms (Li X. et al., 2019). Consistently with the previous study, Li X. et al. showed that PDT mediated by EDTA promoted the formation of bacteria-free voids. In combination with antibiotics, a higher evidence of biofilm destruction was observed, with dead cells present throughout the entire thickness of the treated biofilms and decreased metabolic activities of 8.29% and 7.75% for MRSA and MRPA, respectively (Li X. et al., 2019). Applying a nanotechnological perspective, Darabpour et al. investigated the potentiality of a mixture of polycationic chitosan NPs and methylene blue on the PDT antibiofilm efficiency against 24 h-old *S. aureus* and *P. aeruginosa*. The high affinity of the polycationic chitosan NPs to the negatively charged EPS compromised the adhesion, leading to the disruption of the biofilm (Darabpour et al., 2016). Using carbon nanotubes as a support nanostructure for cationic dye malachite green, Anju et al. proved that exopolysaccharide inhibition (57.84% for *S. aureus* and 37.25% for *P. aeruginosa*) and considerable biofilm reduction can also be achieved in *P. aeruginosa* and *S. aureus* biofilms (Anju et al., 2019b).

As known, bacteria tend to colonize and adhere to several medical devices, such as implants, which represents a huge concern. Considering this, great efforts have been made to design biocompatible and non-invasive phototherapeutic strategies, for instance, based on the coating of titanium implants and near-infrared light (Li M. et al., 2019; Yuan et al., 2019). Promising eradication results have been achieved for *S. aureus* biofilms by taking advantage of the synergistic effect of the ROS produced by PDT and the thermal effect of the photothermal therapy (PTT), without showing noticeable toxicity (Li M. et al., 2019; Yuan et al., 2019). PDT was also studied for prosthetic joint infections, as a mean to target the biofilms that cause these infections (Vassena et al., 2014; Briggs et al., 2018). Vassena et al. studied the application of RLP068/Cl as a PS in biofilms of mature MRSA and *P. aeruginosa*. This study revealed that besides the antimicrobial activity, the tested PS also promoted some biofilm disruption along with an estimated decrease of 45% and 38% in *S. aureus* and *P. aeruginosa* biomass, respectively (Vassena et al., 2014). Briggs et al. explored the effectiveness of methylene blue as a PS, growing bacteria on both polished titanium alloy and hydroxyapatite-coated disk for 3 days. *S. aureus*, MRSA, *S. epidermidis*, and *P. aeruginosa* biofilms were investigated on the polished surface, while only *P. aeruginosa* was tested in the hydroxyapatite-coated disk. PDT treatment presented significant effect on MRSA and *P. aeruginosa* biofilms and completely eradicated *S. aureus* and *S. epidermidis* biofilms. They also showed that PDT was less effective when eradicating mature bacterial biofilms grown on hydroxyapatite-coated disk. However, with higher laser power, light intensity and exposure time, greater antibiofilm activity can be achieved (Briggs et al., 2018).

As an alternative to PDT, the use of ultrasounds to induce mechanical disruption of the biofilm structure has been also investigated (Table 5). Since the 90's, therapeutic ultrasounds combined with antibiotics have been showing promising results, both *in vitro* and *in vivo*, using low intensity (up to 3 W cm^−2^) and low-frequency (a few 100 kHz−1 MHz) (Qian et al., 1997, 1999; Johnson et al., 1998; Carmen et al., 2004, 2005; Ensing et al., 2005). Even recently, the effect of low-intensity and low-frequency ultrasounds is still under investigation, for instance in combination with tobramycin, against beta-lactamases *E. coli* biofilms. It was found that the morphology of the biofilms subjected to the treatment was seriously affected, presenting reduced thickness and a loosened structure. As a consequence, the penetration of the antibiotic increased, which led to an increased antibacterial effect (Hou et al., 2019).

**Table 5:** Biofilm disruptive strategies based on ultrasounds and laser-induced microbubbles.

**Strategy**	**MB/NB characteristics**	**Additional compounds**	**Parameters**	**Bacterial strains**	**Mechanism of action**	**References**
Low-intensity and low-frequency ultrasound	-	Vancomycin (50 mg kg^−1^)	**Frequency**: 28.5 kHz **Duty cycle**: 1:3 **Power density**: 500 mW cm^−2^ **Time of exposure**: 24 or 48 h	*S. epidermidis* (ATCC 35984)	*In vivo* enhancement of antibiotic action	Carmen et al., 2004
	-	Gentamicin (8 mg kg^−1^)	**Frequency:** 28.5 kHz **Duty cycle:** 1:3 **Power density:** 500 mW cm^−2^ **Time of exposure:** 24 or 48 h	*E. coli* (ATCC 10798) *P. aeruginosa* (ATCC 27853)	*In vivo* enhancement of antibiotic action	Carmen et al., 2005
	-	Gentamicin (8 mg kg^−1^)	**Frequency:** 28.48 kHz **Power density:** 500 mW cm^−2^ **Time of exposure:** 24 to 72 h	*E. coli* (ATCC 10798)	*In vivo* enhancement of antibiotic action (applied locally or systemically)	Ensing et al., 2005
	-	Tobramycin (8 and 80 μg ml^−1^)	**Frequency:** 42 kHz **Power density:** 0.66 W cm^−2^ **Time of exposure:** 30 min	ESBLs *E.coli* (Clinic isolates)	Reduction of the biofilm thickness Loss of structure Enhanced of antibiotic penetration	Hou et al., 2019
USMB	SonoVue 30% (v/v)	Vancomycin (100 μg ml^−1^)	**Frequency:** 0.08 MHz **Power density:** 1.0 W cm^−2^ **Duty cycle:** 50% **Time of exposure:** 10 min	*S. epidermidis* (ATCC 35984)	Formation of micropores Reduction of the biofilm density Increased antibiotic penetration	He et al., 2011
	1% and 4% (v/v)	Vancomycin (32 mg l^−1^)	**Frequency:** 300 kHz **Power density:** 0.5 W cm^−2^ **Duty cycle:** 50% **Time of exposure:** 5 min	*S. epidermidis* (ATCC 35984)	Formation of micropores Reduction of biofilm thickness Enhancement of susceptibility	Dong et al., 2013
	1% and 4% (v/v)	Vancomycin (32 mg l^−1^)	**Frequency:** 1 MHz pulsed US waves **Power density:** 0.5 W cm^−2^ **Duty cycle:** 50% **Time of exposure:** 5 min	*S. epidermidis (*ATCC 35984)	Downregulation of the expression of icaA Interference with *quorum sensing* regulator genes	Dong et al., 2017
	1% (v/v) 4–6 μm	Vancomycin (25 mg kg^−1^)	**Frequency**: 300 kHz **Power density**: 0.5 W cm^−2^ **Duty cycle**: 50% **Time of exposure**: 24 to 72 h after surgery; 5 min, 2 times a day	*S. epidermidis* (ATCC 35984)	*In vivo:* Reduction of biofilm thickness Enhancement of susceptibility	Dong et al., 2018
	Perflutren lipid-coated microspheres filled with octafluoropropane gas (mean diameter: 1.1–3.3 μm)	Gentamicin sulfate (50 mg l^−1^); Streptomycin sulfate (50 mg l^−1^)	500 kHz at a peak negative pressure of 1.1 MPa; 16 cycle tone burst; **Frequency:** 1 kHz pulse repetition **Time of exposure:** 5 min	*P. aeruginosa* (PAO1)	Formation of craters Synergistic effect with the antibiotics	Ronan et al., 2016
Laser-induced vapor nanobubbles	NB produced by the laser thermal effect on 70 nm AuNP (1.4 × 10^10^ AuNP ml^−1^)	Pvp-I (0.01%) Chx (0.04%), BzCl (0.06%); Cetr (0.15%), Mupi (0.01%)	**WL:** 561 nm **Fluence:** 1.7 J cm^−2^ **Laser beam diameter:** ~150 μm **Time of exposure:** 1 or 2 laser pulses for 7 ns	*P. aeruginosa* (LMG 27622) *S. aureus* (Mu50)	Enhanced penetration of antibiotics Biofilm disruption	Teirlinck et al., 2019

*AuNP, silver nanoparticles; BzCl, benzalkonium chloride; Cetr, cetrimonium bromide; Chx, chlorhexidine; E. coli, Escherichia coli; ESBLs, extended-spectrum beta-lactamases; MB, microbubbles; Mupi, mupirocin; NB, nanobubbles; P. aeruginosa, Pseudomonas aeruginosa; Pvp-I, povidone-iodine; S. aureus, Staphylococcus aureus; S. epidermidis, Staphylococcus epidermidis; US, ultrasound; USMB, ultrasound and microbubbles; WL, wavelenght*.

With the aim to achieve greater effects, gas-filled particles, called microbubbles have been associated with ultrasounds. Microbubbles provide nuclei for inertial cavitation and lower the threshold for ultrasounds-induced cavitation, resulting in a substantial effect with a reduced exposure time (He et al., 2011; Dong et al., 2017). He et al. showed that the application of ultrasound-targeted microbubbles (USMB) destruction could significantly improve vancomycin activity against 12 h-old *S. epidermidis* biofilms. Ultrasounds-activated sulfur hexafluoride microbubbles and vancomycin created micropores within the biofilm architecture, which facilitated the vancomycin penetration (He et al., 2011). Besides, a significant decrease in the number of viable cells (7.17 log_10_ CFU ml^−1^) was observed, compared to an untreated control (10.51 log_10_ CFU ml^−1^). *In vivo S. epidermidis* biofilms were grown on polyethylene disks and further subcutaneously implanted in rabbit models. The results revealed a significant decrease of the log_10_ numbers of viable CFU cm^−2^ in biofilms treated with vancomycin and ultrasounds, alone or in combination with microbubbles. It is worth to note that the lowest number of viable cells was observed when microbubbles were added to the treatment (He et al., 2011).

The effect of USMB was also investigated by Dong et al. In this study, low-frequency ultrasounds (300 kHz) combined with vancomycin and microbubbles were evaluated in 24 h-old *S. epidermidis* biofilms. Once more, a reduction of the biofilm thickness was observed, which allowed vancomycin to reach inner layers (Dong et al., 2013). Although the biophysical effect of acoustically activated microbubbles is evident, the underlying mechanisms of interaction between the bubbles and the biofilm has not been elucidated yet (Dong et al., 2017). Therefore, besides the mechanical effects of the USMB treatment in combination with vancomycin, Dong et al. showed that ultrasonic energy could also present biochemical effects on extracellular matrix of *S. epidermidis* biofilms. An ultrasounds treatment (1 MHz, 0.5 W cm^−2^, 50% duty cycle, for 5 min) combined with microbubbles interfered with *quorum sensing* regulator genes and reduced the expression level of icaA, which is one of the encoding genes to polysaccharide intercellular adhesin, a major component of *S. epidermidis* EPS matrix. Although biochemical effects could not be completely distinguished from the mechanical effects of ultrasounds, the combination of all mechanisms resulted in a reduction of the biomass and an enhanced antibiotic activity, through fragilization of the matrix (Dong et al., 2017). As a continuum of the previous studies and with the aim of facilitate the translation of this technology, Dong et al. further explored this same synergistic effect in an *in vivo* rabbit model (Dong et al., 2018). Consistently, SEM images showed that when treated with USMB in combination with vancomycin, biofilms presented the greatest reduction in terms of thickness as well as bacterial density, compared to the other treatment groups. The susceptibility to the antibiotic significantly increased, since the reduction of bacterial counts with USMB + vancomycin treatment was close to three orders of magnitude compared with the control. Also, histopathologic examinations showed no damage to the skin and organs, as a consequence of ultrasounds alone or USMB, which indicates that these technologies may be well tolerated by the body and have the potential to be safely applied (Dong et al., 2018).

Other conventional antibiotics, such as gentamicin and streptomycin, have also been tested in association with USMB (Ronan et al., 2016). It was found that exposing 72 h-old *P. aeruginosa* biofilms to USMB alone caused significant structural damage to the biofilm, through the formation of voids and 5–20 μm diameter craters. The ultrasonic disruption of the microbubbles enables the resulting shockwaves and microjets to act nearby the biofilm, affecting not only the permeability of the cells but also the EPS matrix integrity. The additional action with the antibiotics led to a significant reduction in overall biomass and thickness of the treated biofilms as well as a reduction in the metabolic activity of the bacteria, detected by CO_2_ production rate quantification (Ronan et al., 2016).

Besides ultrasounds, laser light can also be used to induce the vaporization of nanobubbles, producing similar damage on the matrix and potentiating antibiotic effects (Teirlinck et al., 2019). Teirlinck et al. tested this approach against *P. aeruginosa* and *S. aureus* biofilms, in conjugation with small gold NPs. When short laser pulses were applied to the biofilms, these NPs could absorb the energy of high intensity, heating the water surrounding of the particles that quickly evaporated, resulting in expanding and imploding water vapor nanobubbles (Teirlinck et al., 2019). The organized structure of the biofilm was compromised by the generated pressure waves, enabling a better diffusion of the drug molecules deep into the biofilm. In fact, the treatment with vapor nanobubbles enhanced the antibacterial effects of the tested compounds, achieving results comparable with the ones obtained for forced disrupted biofilms, by sonication and vortexing (Teirlinck et al., 2019).

Throughout this section, it was shown that both PDT and ultrasounds are efficient technologies for the eradication of biofilms. Niavarzi et al. recently considered the hypothesis of combining these two strategies, by using ultrasounds to activate a PS (methylene blue), followed by PDT. In fact, ultrasonic activation of PS in conjugation with the PDT increased the penetration depth into *E. faecalis* biofilms, leading to greater antibiofilm activity of the PS compared to the use of PDT alone, with reduction bacterial counts of ~98%, comparing to the control group (Niavarzi et al., 2019).

## Future Perspectives

Biofilm-associated infections are nowadays a major concern for the healthcare systems. At a clinical level, the current therapies available are mainly focused only on a biocidal approach. Strategies focusing on targeting and disruption of the EPS matrix may be a promising approach to increase cell susceptibility to antibacterial agents (Fulaz et al., 2019). Antibiofilm agents have been reported in the literature to potentiate the activity of antibiotics due to their ability to disassemble the matrix. In a more complex strategy, NPs as carriers of antibiofilm agents have been developed. However, these nanocarriers are mostly focused on the immobilization of matrix disruptive enzymes, such as DNase I, dispersin B, and alginate lyase. To the best of our knowledge, nanocarriers for delivery of mucolytic agents with antibiofilm potential have not been developed.

Regarding the explored innovative technologies, it was shown that physical disturbance of the EPS matrix is also achieved, through generation of ROS, interference with matrix polysaccharides or creation of voids and craters in the biofilm structure. Although these mechanisms can efficiently disrupt biofilms without development of bacterial resistance, cell detachment can increase the risk of reinfection. Thus, a synergistic effect between the antibacterial effect of antibiotics and the mechanical disruption caused by these technologies is aimed in most reported studies.

In conclusion, the ideal strategy for biofilm eradication would consider the combination of antibacterial agents with strategies for EPS disassembly. In this scenario, the EPS would be disrupted, and the dispersed bacterial cells would be further killed by a bactericidal agent. Hence, this innovative therapy would decrease bacterial resistance and prevent biofilm recurrence.

## Author Contributions

RP and FS drafted the manuscript. SR, CN, and PV reviewed and edited the manuscript. All authors read and approved the final version.

## Conflict of Interest

The authors declare that the research was conducted in the absence of any commercial or financial relationships that could be construed as a potential conflict of interest.

## Abbreviations

AC: altering current
AgNPs: silver nanoparticles
ALA: 5-aminolevulinic acid
APTES: 3-aminopropyltriethoxysilane
C_2_DA: *Cis*-2-decenoic acid
CES: carboxyethylsilanetriol
CLSM: confocal laser scanning microscopy
CS: chitosan
DC: direct current
DNAse I: deoxyribonuclease I
*E. coli*: *Escherichia coli*
eDNA: extracellular DNA
EDTA: ethylenediaminetetraacetic acid
*E. faecalis*: *Enterococcus faecalis*
*E. faecium*: *Enterococcus faecium*
EOs: essential oils
EPS: extracellular polymeric substances
MBEC: minimum biofilm eliminating concentration
MIC: minimun inhibitory concentration
MNPs: magnetic nanoparticles
MOFs: metal organic frameworks
MRPA: multidrug-resistant *Pseudomonas aeruginosa*
MRSA: methicillin-resistant *Staphylococcus aureus*
NAC: *N*-acetyl cysteine
NPs: nanoparticles
PAE: poly(β-amino ester)
*P. aeruginosa*: *Pseudomonas aeruginosa*
PAO1: *Pseudomonas aeruginosa* O1
PDT: photodynamic therapy
PEG: polyethylene glycol
PNAG: poly-*N*-acetylglucosamine
PS: photosensitizer
PTT: photothermal therapy
ROS: reactive oxygen species
SEM: scanning electron microscopy
SPIONs: superparamagnetic iron oxide nanoparticles
*S. epidermidis*: *Staphylococcus epidermidis*
*S. aureus*: *Staphylococcus aureus*
TMP: tetra-substituted *N*-methyl-pyridyl-porphine
USMB: ultrasound-targeted microbubble.
